# Cyclic Fatigue Resistance of Glide Path Rotary Files: A Systematic Review of in Vitro Studies

**DOI:** 10.3390/ma15196662

**Published:** 2022-09-26

**Authors:** Israa Ashkar, José Luis Sanz, Leopoldo Forner

**Affiliations:** Department of Stomatology, Faculty of Medicine and Dentistry, Universitat de València, 46010 Valencia, Spain

**Keywords:** glide path, patency, rotary files, cyclic fatigue, systematic review

## Abstract

The aim of the present systematic review was to perform a qualitative synthesis of in vitro studies that assess the cyclic fatigue resistance of rotary glide path (GP) files of endodontic applications. Systematic electronic searches were performed in the Medline, Embase, Scopus, SciELO, and Web of Science databases on 15 February 2022, and were last updated on 1 April In vitro studies that evaluated and compared the cyclic fatigue resistance of at least one rotary GP file system with another rotary GP file system were included. A total of 25 studies were included in the qualitative synthesis. All studies assessing the difference in the cyclic fatigue resistance between continuous and reciprocating rotation in rotary glide path files found that the latter resulted in a significantly higher cyclic fatigue resistance, as evidenced by a higher number of cycles until fracture and/or time until fracture. Within the limitations of this review and the in vitro nature of the included studies, the results indicate that the cyclic fatigue resistance of rotary GP files may be influenced by several intrinsic factors of the files, such as their taper, cross-sectional design, alloy properties, kinematics, and external factors, such as the curvature and radius at which the file is activated, the irrigation or lubricant used, and the temperature.

## 1. Introduction

An adequate tridimensional seal of a root canal can be achieved by establishing a preliminary glide path (GP) that can subsequently aid the instrumentation or shaping process. GP preparation results in an unimpeded radicular tunnel running from the canal orifice to the physiological terminus, with a minimal size of a loose ISO 10 endodontic file inside the canal [1]. Creating a GP is a preliminary step during root canal treatment (RCT), and is performed prior to the use of rotary files of greater tapers inside the root canal [2]. Its creation before instrumentation is a recommended clinical step to facilitate the penetration of subsequent rotary files, regardless of the technique used. GPs maintain the original shape of a root canal and improve the effectiveness of the shaping procedure, preventing the file from fracturing and preventing iatrogenic events such as the formation of ledges, perforations, or blockages of the canal [2]. Furthermore, it has been reported that forming a GP prior to the main instrumentation process results in a significant decrease in postoperative pain, which may result from the amount of apical debris extrusion [3].

Several instruments have been proposed for the creation of an adequate GP, such as manual stainless steel (SS) K-files, the combination of SS K-files and a reciprocating handpiece, and nickel titanium (NiTi) rotary files.

Manual SS K-files present a constant taper of 2% and were the first to be proposed as GP files for RCT. Classically, West [1] recommended the use of a manual file of at least an ISO 10 size to create GPs by following and identifying the entrance to the canal and removing any dentin tissue or debris that could hinder the access to the canal in a straight path. This is performed by slightly pre-curving the last apical millimeters of the smallest file that easily fits inside the canal using metal cotton pliers. Finally, with multiple vertical strokes of short amplitude, the file must be loose inside the canal [1].

The use of SS manual GP files presents several advantages, such as an increased tactile sensation, the maintenance of the anatomical curvatures and eccentricities of the canal, a lower potential for fractures inside the canal, a high ability to bypass an intracanal blockage or obstacle, and a reasonable cost [4]. However, creating GPs with SS manual files is time consuming [5] and presents a greater risk of iatrogenic occurrences such as perforations and transportation [6], which have caused its use to be diminished among clinicians.

Reciprocating file movement, however, is known to reduce torsional stress and fatigue in endodontic files [7]. Accordingly, the use of SS K-files inserted into a reciprocating handpiece has appeared as a low-cost alternative to the use of the rotary files and as a solution to overcome some of the limitations of the use of manual files to create a GP. The use of this technique for GP creation reduces the preparation time and the operator’s hand fatigue compared to the conventional manual technique, while reducing the risk of intracanal instrument separation compared to rotary NiTi files [8]. However, the complications associated with the use of SS files in a reciprocating handpiece, such as apical transportation with files larger than a 15 K-File [9] and excess dentine removal [10], are some of the reasons for the lack of use of this technique among clinicians.

The introduction of NiTi alloys into the endodontic field and the subsequent automation of the process of mechanical preparation using rotary systems were the beginning of a new generation of instruments for RCT [11]. These new systems exhibited notable progress in terms of shaping: they increased the predictability in root canal shaping due to their flexibility—especially with respect to their use in curved root canals [12], the decreased modification of root canal curvature and anatomy in comparison with manual files [13], and a reduction in working time, which provides more comfort for both clinicians and patients. NiTi alloy exhibits a high biocompatibility and corrosion resistance due to its titanium oxide surface coating [14]. In addition, the superplasticity of NiTi alloys is associated with substantial recoverable deformation when exposed to load and discharge at a suitable temperature [14].

In the late 1980s, Walia, Brantley, and Gerstein introduced the first NiTi endodontic instruments manufactured by conventional machining [15], and they were used until 1999, when NiTi alloy treatments were introduced and drastically changed their clinical behavior. Currently, instruments are manufactured from different NiTi alloys that are exposed to different treatments [16], with superelastic properties and shape memory [15]; they use centric or eccentric movement in either rotational or reciprocating kinetics. Currently, GP preparation is achieved via the preliminary use of patency hand files to negotiate along the root canal, followed by the use of rotary NiTi GP files that have shifted over the years from multi-file systems into two-file and single-file systems.

Despite the various advantages and the superelasticity of these rotary files, the risk of fracture within the canal remains a relevant concern when creating GPs, especially in severely curved root canals where the instrument encounters high stress or compression while rotating at the point of the greatest curvature [17]. File fracture can occur through two mechanical mechanisms: torsional fatigue, which can occur when the instrument rotates while binding within the canal, and cyclic fatigue, which occurs when the metal is exposed to repetitive compaction and traction forces that cause the deterioration of the file structure and can eventually lead to fracture [7].

To date, no reviews have been performed to study the cyclic fatigue resistance of rotary GP files. Therefore, the present systematic review aims to present a qualitative synthesis of the available scientific literature consisting of in vitro studies that have evaluated the cyclic fatigue resistance (CFR) of rotary GP files.

## 2. Materials and Methods

### 2.1. Protocol and Registration

The present work followed the guidelines recommended by the PRISMA 2020 Statement (Preferred Reporting Items for Systematic Reviews and Meta-analysis) [18]. The systematic review protocol was previously registered and is available in the Open Science Framework (OSF) repository (DOI 10.17605/OSF.IO/6KVEP).

### 2.2. Elegibility Criteria

In vitro studies that compared two or more rotary GP file systems were eligible. Studies assessing only one GP file system were excluded. The inclusion criteria were based on the PICOS framework [19], as follows: population/problem (P): rotary GP files, intervention (I): cyclic fatigue test, comparison (C): commercially available or experimental rotary GP files, outcome (O): cyclic fatigue resistance, study design (S): in vitro studies.

### 2.3. Search Strategy and Terminology

The search process, study selection, data extraction, and quality assessment were carried out by two independent examiners (I.A. and J.L.S). A third author was consulted in the event of any discrepancy between them (L.F.).

A systematic electronic search was carried out in two stages, as explained below:

First search: An advanced electronic search was performed in the Medline (via PubMed), Embase, Scopus, SciELO, and WOS databases on 15 February 2022, and was last updated on 1 April 2022, without any years of publication or language restrictions. The following keywords were used: “glide path”, “patency”, “rotary files”, “root canal”, “endodod *”, “cyclic fatigue”, and “fracture”. The Boolean operators “AND” and “OR” were used to annex the terms and develop the search strategy. The full search strategy is available in Supplementary Table S1.

Second search: Additional specific searches were performed in the aforementioned databases on 15 February 2022, and were last updated on 1 April 2022, without restrictions. The names of the different commercially available files that were assessed by the selected studies resulting from the first search were used as search terms.

Additionally, the references of the included studies were screened for potentially eligible studies that did not appear in the preliminary database search.

### 2.4. Study Selection Process

All references identified using the search strategy were exported from each database into Mendeley reference manager software (Elsevier, Amsterdam, The Netherlands), and duplicate records were discarded manually. Subsequently, an initial screening of the titles and abstracts of the resulting records was performed. Studies that met the inclusion criteria in the first screening were then retrieved and an assessment of their full texts was performed to confirm their eligibility.

### 2.5. Data Extraction Process

The data synthesis of the included studies was divided into variables for study characteristics, methodology, and results. The variables extracted for study characteristics were: author and year of publication. The variables for study methodology included: rotary GP files assessed, total/group sample size, canal anatomy, curvature angle of curvature/radius, lubricant, procedure/s used to evaluate the resistance to cyclic fatigue, RPM, and torque.

### 2.6. Quality Assessment

The studies included in this review were independently assessed for internal methodological risk of bias using the “Modified CONSORT checklist of items for reporting in vitro studies of dental materials” [20] by assessing each study’s compliance with each of the parameters or elements considered on the checklist. Once registered, the percentage of compliance for each of the studies was calculated.

## 3. Results

### 3.1. Study Selection

The search identified a total of 381 preliminary results: 145 articles were identified through the first search, where 30 were found in PubMed, 12 in Scopus, 24 in Embase, and 79 in WOS. The search in SciELO yielded no results. A total of 236 articles were identified through the complementary searches for each file (ProGlider: 63; Pathfile: 65; R-Pilot: 35; WaveOne Gold Glider: 22; OneG file: 11; G File 11; Hyflex file and glidepath: 16; ScoutRaCe: 6; Mtwo file: 6; Race ISO 10 file: 5; EdgeOne Fire GlidePath: 0; and V glide path: 2.

**Figure 1:**
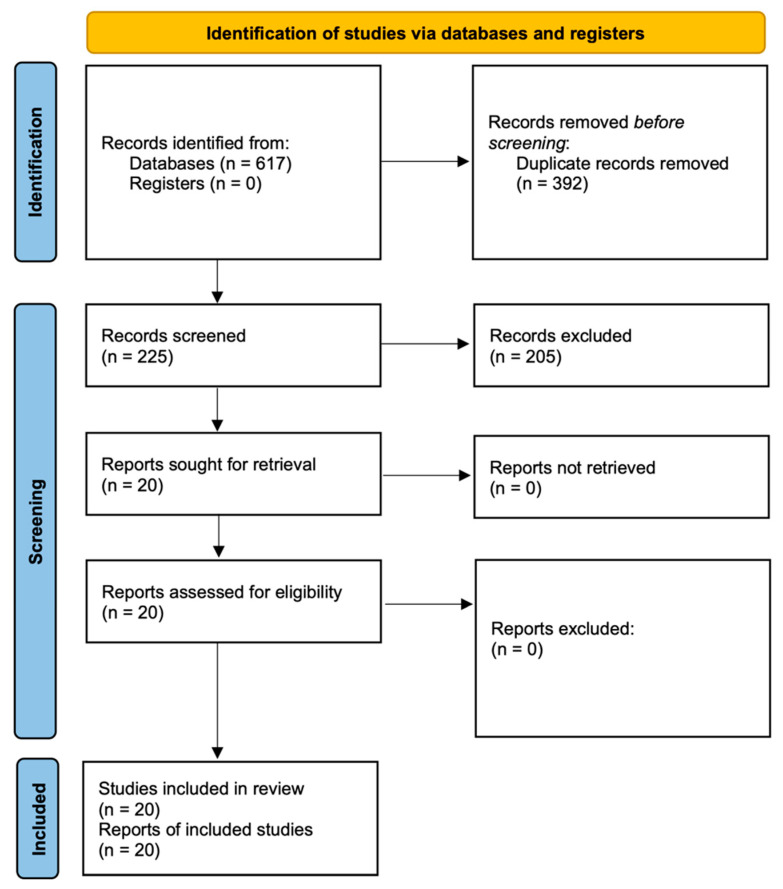
Systematic flow chart representing the study selection process, based on the PRISMA 2020 flow diagram [18].

Duplicates were manually removed using Mendeley reference management software (Elsevier, AMS, Netherlands), resulting in 225 records. From there, 205 records were excluded upon screening the title and abstract. The 20 resulting articles were evaluated by reading the full text, and all of them were considered eligible for qualitative synthesis [21,22,23,24,25,26,27,28,29,30,31,32,33,34,35,36,37,38,39,40] (Figure 1).

### 3.2. Study Characteristics

The list of commercially available glidepath rotary files assessed by the included studies, along with their manufacturers and main characteristics, are presented in Table 1.

The general methodological characteristics of each included study are shown in Table 2. From the study sample (*n* = 20), fifteen studies used stainless steel (SS) artificial canals, three used tampered steel, and one study used a device with three stainless steel pins. The remaining study did not specify the type of artificial canal used. The angle of curvature and radius among the studies varied between 45 and 90 degrees and 2 and 6 mm, respectively. Four of the included studies used double-curved canals, while all others used a single-curved canal.

The sample size varied between two and thirty files per experimental group in each study that evaluated the cyclic fatigue resistance by either assessing the time required for the file to fracture (TF) or the number or cycles the file made before it fractured (NCF).

Most studies used a lubricant to minimize the friction between files and the artificial canal walls, as observed in Table 2: thirteen studies used oil-based lubricants, two used water, one study used NaOCl alone or with HEBP (1-hydroxyethylidene-1,1-bisphosphonate), and one study used glycerin. Three studies did not mention whether a lubricant was used.

**Figure 2:**
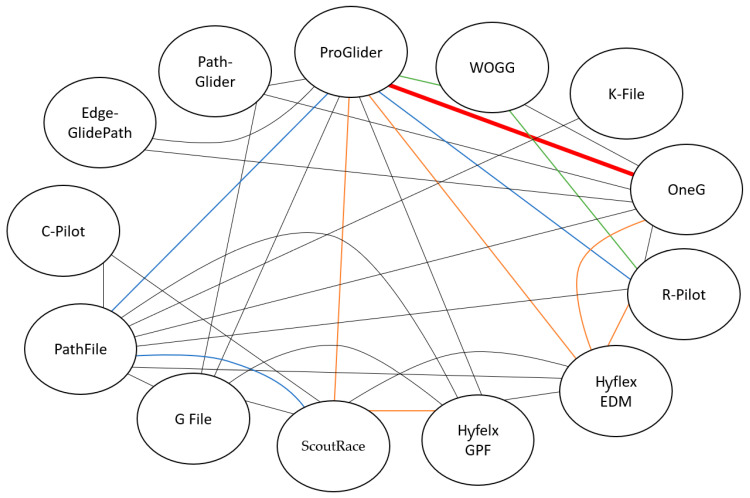
Schematic representation of the number of comparisons between files among the included studies. Color key: red (files are compared 6 times), green (files are compared 4 times), blue (files are compared 3 times), yellow (files are compared 2 times), and black (files are compared once).

Complementarily, the different comparisons made between glidepath rotary files are depicted in Figure 2, where it can be seen that the modal comparison among the study sample was ProGlider vs. OneG (six times), followed by WaveOne Gold Glider vs. R-Pilot (four times) or ProGlider (four times).

### 3.3. Study Results

The significant results reported by the included studies in terms of the number of cycles until failure, the time to fracture, and the length of the fractured fragment are presented in Table 3. Quantitative results with regards to the time until fracture (TF), the number of cycles until fracture (NCF), and the length of the fractured fragment (LF) are presented in Supplementary Table S2.

The R-Pilot and WOGG reciprocating rotary files exhibited a higher CFR in comparison with the continuous rotary files (ProGlider, Hyflex EDM, PathFile, and OneG), either by a significantly higher TF [35] or NCF [30,32,39]. The comparison between both files, however, offered mixed results among studies. R-Pilot exhibited a significantly higher TF than WOGG in one study [33], while WOGG showed a significantly higher CFR than R-Pilot in two other studies [29,30]. In another study, no significant difference was found between the two files in terms of TF [35].

With regards to the most commonly compared files, ProGlider and OneG, ProGlider exhibited a significantly higher CFR than OneG in all cases, as shown by a significantly higher TF [34,38] and NCF [26,28,37,39].

Hyflex EDM also demonstrated a significant higher CFR than the other continuous rotary files: PathFile [32], OneG [28,34], Hyflex GPF [31], RaCe [31], and ProGlider [28].

PathFile underwent a significantly higher number of cycles until fracture than ScoutRaCe in two of the studies [21,25], while it showed a significantly lower CFR than other files: HyFlex GPF [25], G file [25], ProGlider [23,25,36], R-Pilot [32], and HyFlex EDM. In parallel, one of the studies reported that PathFile #1 showed a significantly higher CFR than the rest of the files in its system (PathFile #2 and PathFile #3) [22].

One of the studies evaluated the CFR of two prototype files with or without heat treatment, and with a different pitch length in the files OneG and ProGlider. It reported that the heat-treated files had a higher CFR than the non-heat-treated files. In addition, the files with a shorter pitch showed a higher CFR than the files with a longer pitch, regardless of whether the files were heat-treated or not [27].

No significant differences were found regarding the length of the fractured fragments.

### 3.4. Quality Assessment

The results of the evaluation of the quality of evidence using a modified CONSORT checklist for the quality assessment of the in vitro studies on dental materials [20] are presented in Table 4.

The mean compliance of the included studies was 72%, with a maximum score of 82% and a minimum score of 55%. All the in vitro studies included in this review presented a structured abstract (item 1) and an introduction that provided information on the history of the GP files and their CFR (item 2a). Within the introduction, all studies presented the objectives and hypotheses of the study (item 2b).

The description of the methodology, as well as the variables studied, was clear enough to allow its replication in all studies (items 3 and 4). Item 10, indicating the statistical methods used to compare the groups, was also met by all included studies. Items 6–9, which refer to the randomization process, were not applicable to these studies. Lastly, no reference to the available full trial protocols was made in any of the studies (item 14).

The differences between the studies were in items 5 and 11–13 which indicated how the sample size was determined in the methods, the outcomes and estimation in results, whether the trial limitations were mentioned in the discussion, and whether the source of funding was mentioned at the end of the study, respectively.

## 4. Discussion

Creating a glidepath using NiTi rotary files is recommended to achieve a more predictable preparation compared to manual files. The desirable mechanical properties of NiTi alloys facilitate the preparation of root canals within a shorter time, produce less canal transportation, allow for a higher dentin preservation, and have a reduced risk of apical deformation or zipping in curved canals [41,42,43]. However, the fracture of rotary NiTi GP files is still a major drawback that must be considered in clinical practice. Accordingly, the aim of this study was to present a systematic review of the available literature investigating the CFR of rotary GP files with in vitro studies.

Previous studies have demonstrated that reciprocating rotary files evidence a higher CFR compared to continuous rotary files [7,44,45]. In this systematic review, it was observed that GP reciprocating rotary files also exhibited a higher resistance to cyclic fatigue fracture than the continuous rotation files [30,32,34,35,39]. In fact, one of the included studies compared the CFR of PathFile with a manual K-file attached to a M4 reciprocating handpiece (SybronEndo, Glendora, CA, USA), and reported that even stainless-steel K-files showed a significantly higher TF than PathFile [24], although both files had similar design features. This suggests the important role that reciprocating movement plays in the increase of the CFR of endodontic instruments, regardless of alloy of which the file is made.

However, three of the included studies [30,32,39] compared the CFR of reciprocating files in terms of the NCF. According to previous studies [46,47,48], NCF is not an ideal indicator of CFR for two main reasons: (1) it is not possible to establish this parameter with precision for instruments that make forward and backward angles, and (2) the real kinematics of reciprocating endodontic motors differ from the established values declared by the manufacturer. This aspect should be taken into consideration in future investigations.

A clear heterogeneity among the methodology of the included studies was observed. Some studies activated the files in static mode [22,32,33,40] while other studies activated them in dynamic mode [26,29,35,36,37,39] until fracture. Previous studies have reported that dynamic pecking motion tests are best suited for cyclic fatigue investigations when using NiTi rotary files, as static tests cannot reproduce the actual conditions encountered in clinical practice [49,50]. Automated instrumentation systems have been designed to prepare root canals in a dynamic movement with specific values of torque and speed, in order to extend the useful life of files. Therefore, the surface tension caused by the continuous stress used in static motion models is not representative of the clinical use of the files [51]. In addition, the peck amplitude provides a time interval before the instruments are again subjected to the area of greatest stress. The pecking motion can be a fundamental factor in the prevention of the fracture of the NiTi rotary files to the extent that it minimizes the stress on the files in curved canals. This decreases the possibility of fracture while remaining at a single depth in the canal, which will favor the premature damage of the instruments [52].

Natural teeth are the best specimens in which to evaluate the CFR of NiTi rotary files, but it is difficult to achieve uniformity in terms of canal length, degree, the radius of curvature, and even dentine hardness [53]. However, all the included studies in this review used artificial canals to perform the CFR test on the files. The majority used SS [21,24,25,26,29,30,32,33,34,35,36,37,38,39,40], some used tampered steel [22,23,27], and another study used an SS 3-pin device [31]. One study did not specify the type of artificial canals used [28]. Artificial canals are used to standardize the canals in a desired shape or curvature, allowing researchers to overcome the differences imposed by the various variables inherent in extracted teeth. This allows researchers to perform accurate and reproducible evaluations of the mechanical properties of files [54]. Nevertheless, performing CFR tests in such canals does not reflect clinical conditions such as the dentin microhardness [40]. For this reason, the results of studies that use artificial canals should be extrapolated into clinical practice with caution [55].

With in vitro studies, lubricants are used to allow free motion of the instrument, to minimize the release of heat, and to minimize friction between the file and the walls of the artificial canal. Most of the included studies used synthetic lubricants, while others used water, glycerin, or NaOCl with/without HEBP. However, except for NaOCl, the aforementioned lubricants are not normally used in daily clinical practice, as they lack any tissue-dissolving or antimicrobial effects, which are important features of irrigating solutions in root canal treatments [56]. For this reason, using actual root canal irrigation solutions such as NaOCl in similar future studies appears to be more suitable to better extrapolate the results into the clinical setting [57].

The CFR of the tested files was also affected by the different canal curvatures, the radii used in each study, and the location of the curvature. For example, it was observed that all the tested instruments in one study had a lower NCF at the 3 mm curvature radius when compared with the 5 mm curvature radius [25]. It was also observed that the files had less CFR in terms of both TF and NCF in increased curvatures [29]. In addition, it was found that all studies that used double-curvature canals resulted in the files breaking within less time and after a smaller number of cycles in apical curves than in coronal ones, exhibiting a higher CFR in coronal curvatures [28,32,36,39].

Three of the included studies [33,34,40] performed the CFR analysis at body temperature or intracanal temperature (35 degrees approximately) to better simulate the clinical conditions when establishing a glide path in a root canal. It is worth mentioning that a previous study [58] reported that cooling down files to a low temperature during the instrumentation process, albeit difficult, actually improves the fatigue resistance of both heat-treated and conventional rotary NiTi files. This is related to the relative amount of the martensitic phase of the file, which seems to determine the NCF. From a metallurgic perspective, the austenite start (As) and austenite finish (Af) temperatures (which indicate the temperatures at which the change in the file’s metal structure from martensite to austenite starts and completes, respectively) are increased by heat treatments [59]. When the temperature is between (As) and (Af), the alloy consists of both austenite and martensite phases, exhibiting a higher fatigue resistance [60]. When the alloy cools down to a temperature close to (As), the percentage of the martensitic phase could reach the maximum range possible for the alloy itself [61]. In other words, the cyclic fatigue resistance of these files is reduced when the temperature increases. The different reactions of alloys to different temperatures make it critical to include the simulation of environmental conditions such as body or intercanal temperature when performing cyclic fatigue tests on rotary files.

Apart from the differences in the methodologies of each included study, several factors related to the files themselves may have influenced their flexibility and cyclic life span, such as their cross-sectional design [62], the type of metal alloy and the alloy’s treatment [63,64], and the size and taper of the file [65]. In future studies, it could be interesting to perform comparisons from the perspective of a specific property of the files.

Among the included studies, ProGlider was the modal file assessed and compared (Figure 2). This M-wire alloy file exhibited a significantly higher CFR compared to conventional NiTi files [23,26,36,38], but showed a significantly lower CFR compared to HyFlex GPF, Hyflex EDM, and EdgeGlide Path files [25,28,37]. This could be attributed to the differences in their cross-sectional design or their alloy treatment (Table 1). ProGlider also showed a significantly lower CFR compared to WOGG and R-Pilot [30,34,35,39,40]. This could be due to several reasons—not only the differences in their cross-section or alloy properties, but also the reciprocating kinetics. EdgeGlide Path and PathGlider were only evaluated once [37,38]. This limits the analysis of their CFR and calls out for further studies on their properties.

From a general viewpoint, it was observed that the longest times needed for a fracture to occur for both R-Pilot (3562.46 ± 963.55 s) and WOGG (3465.26 ± 468.54 s) were recorded in a study by Keskin [35], while the shortest TFs registered for these files were found in a study by Topçuoğlu [29] (247.2 ± 36.2 s and 368.3 ± 44.1 s, respectively). This is interesting because both studies had similar characteristics in terms of the lubricant used, the artificial canal type, the angle of curvature/radius of the canal (60°/5 mm), and even the file activation mode, which was dynamic. The temperature of the file’s environment was not considered in any of the studies, which might have had an influence on the outcome. As was reported in a previous study, the temperature at which NiTi files are tested may influence the results of CFR in a different manner depending on the heat treatment of the alloy [66].

On another note, ProGlider´s longest TF was 1254.20 ± 356.08 s [35] in a canal of 60° curvature and a 5 mm radius, and its shortest TF was 101 s [38] in a canal of 90° curvature and a 3 mm radius. Likewise, these two studies had similar characteristics except for the canal´s curvature and radius. This shows the important effect of the angle of curvature and its radius on the CFR.

Hyflex EDM also exhibited a different CFR in different studies. Its shortest TF was 388.21 ± 46.62 s [34]. Its longest TF, however, was registered in a study by Nishijo 36, which activated the files in continuous rotation and in a reciprocating motion to study the effect of the kinetics on the files. Hyflex EDM´s TF was 700 s in a continuous rotation and 1650 s in a reciprocating motion. These differences may be due to the different lubricants and artificial canals used. Yılmaz [34] used water as lubricant and an SS artificial canal, while Nishijo [31] used an SS 3-pin device and silicone oil, which may have positively affected the CFR of the files.

Among the studies that evaluated the NCF of the files, it was noticed that the canal´s curvature and its radius were the main common variables that influenced the CFR of the files. This was observed with ProGlider, as its highest NCF was 25.082 ± 6009.37 [26] in a canal of a 60° curvature and a 5 mm radius, while the lowest was 266 ± 72 [30] in a canal with a 90° curvature and a 3 mm radius, considering that other variables such as the lubricant and canal type were the same. The highest NCF for WOGG that was registered was 2304.36 ± 346.21 in a 45° curved canal and a 5 mm radius [29], while the lowest was 1294 ± 123 in a (90°/3 mm) canal [30]. R-Pilot´s lowest NCF was 1038 ±177 in a 90°/3 mm canal [30], while the highest NCF was 4894.82 ± 743.11, which was registered in a coronal curve (60°/5 mm) of a double-curved SS canal [32]. The same was found for PathFile: its highest NCF was reported in the coronal curve (60°/5 mm) of a double-curved canal [32] and the lowest was registered in a single curved canal with a 90°/5 mm angle and radius, respectively [25]. Altogether, the results indicate that the more severe the canal curvature and the smaller its radius, the less resistance the file has towards cyclic fatigue.

With regards to the quality assessment of the included studies, their overall designs reported a structured abstract, straightforward aims or hypotheses, an exhaustive and detailed description of the methodology to allow for replication, and relevant conclusions. However, most did not acknowledge the process used to determine the sample size and failed to address the potential limitations of their studies in the discussion section. In addition, the majority did not mention whether there had been a source of funding or other support and none of the authors mentioned where a full trial protocol can be accessed or if it was available. Items 6–9 were considered as non-applicable, since they were related to the randomization process.

Considering the average compliance of the studies with quality assessment parameters and the in vitro nature of the performed assays, the results of the studies and, consequently, of the present systematic review should be interpreted with caution, since the laboratory-based results may not reflect real clinical behavior. This acts as a limitation of this review. Another limitation of this review is the heterogeneity in the methodologies and the files used in the included studies, which made it impracticable to carry out a quantitative analysis or meta-analysis and to reach more specific conclusions. Nevertheless, the qualitative synthesis performed in the present review elucidates the importance of the type of motion (reciprocating or continuous) in the CFR of rotary GP files. Future studies in the field could compare the CFR between different types of files, such as rotary GP files and shaping files. From a clinical perspective, clinicians should be aware of the characteristics of rotary GP files in order to select the most appropriate files for specific cases and know their limitations in terms of CFR.

## 5. Conclusions

Within the limitations of this review and the in vitro nature of the included studies, the results indicate that the cyclic fatigue resistance of rotary GP files may be influenced by several intrinsic factors of the files, such as their taper, cross-sectional design, alloy properties, and kinematics.

External factors can also influence the cyclic fatigue resistance of files, such as the curvature and radius at which the file is activated, the irrigation or lubricant used, and the temperature.

All studies assessing the difference in cyclic fatigue resistance between continuous rotation and reciprocating movement in glide path rotary files found that the latter resulted in a significantly higher cyclic fatigue resistance, as evidenced by a higher number of cycles until fracture and/or time until fracture.

## Figures and Tables

**Table 1:** List of commercially available rotary GP files included in this study.

File	Manufacturer Data	N° of Instruments	Alloy	Diameter; Taper	Length (mm)	Cross-Section	Kinematics
ProGlider	Denstply Maillefer; Ballaigues, Switzerland	1	M-Wire	16; variable taper (2%)	21, 25, 31	Square	Continuous rotation
PathFile	Dentsply Maillefer; Ballaigues, Switzerland	3	Conventional NiTi	13, 16, 19; (2%)	21, 25, 31	Square	Continuous rotation
ScoutRaCe	FKG Dentaire; La Chaux-de-Fonds, Switzerland	3	Conventional NiTi	10, 15, 20; (2%)	21, 25, 31	Square	Continuous rotation
R pilot	VDW; Munich, Germany	1	M-wire	12.5; variable taper (4%)	21, 25, 31	S-shaped	Reciprocating motion
WaveOne Gold Glider	Dentsply Maillefer; Ballaigues, Switzerland	1	Gold wire	15; variable taper (2%)	21, 25, 31	Parallelogram	Reciprocating motion
OneG	Micro-Mega; BESANCON FRANCE	1	Conventional NiTi	14; (3%)	21, 25, 29	Asymmetrical	Continuous rotation
G file	Micro-Mega; BESANCON FRANCE	2	Conventional NiTi	12, 17; (3%)	21, 25, 29	Square	Continuous rotation
HyFlex GPF Glide Path File	Coltene; Whaledent, Altstatten, Switzerland	3	CM-Wire	15, 20; (2%)	21, 25, 31	Square	Continuous rotation
HyFlex EDM Glide Path File	Coltene; Whaledent, Altstatten, Switzerland	1	CM-Wire, with electrical discharge machining	10; (15%)	21, 25	Square at the tip, trapezoidal in the middle, and triangular towards the axis	Continuous rotation
PathGlider	Komet; Brasseler, Lemgo, Germany	1	Conventional NiTi	15, 20; (2%)	21, 25, 31	Kite shape (asymmetric rhombus)	Continuous rotation
Edge Glide Path	Edge Endo; Albuquerque, NM, USA	2	Heat Treated FireWire ^®^ NiTi	19; variable taper	21,25, 31	Triangular	Continuous rotation

**Table 2:** Study characteristics.

Author, Year	Files (Size/Taper)	Sample Size (Group/Total)	Canal Anatomy	Angle of Curvature/Radius	Lubricant	CFR Assays	RPM/Torque	Temperature at Which Files Are Activated
Lopes et al., 2012 [21]	Motor-driven C-Pilot file (10/0.02); PathFile (13/0.02); ScoutRaCe (10/0.02)	10/30	SS	-/6 mm	Glycerin	NCF FL	C-Pilot: 300 rpm; PathFile: 300 rpm; ScoutRaCe: 300 rpm	
Sung et al., 2014 [22]	G File: G1(#12/0.03), G2(#17/0.03); Path Files: PathFile #1 (#13/0.02), PathFile #2 (#16/0.02), PathFile #3 (#19/0.02)	2/10	Tempered Steel	90°/3 mm	Synthetic oil (WD-40; WD-40 Company, San Diego, CA, USA)	NCF FL	G File: 300 rpm; PathFile: 300 rpm	
Elnaghy and Elsaka, 2015 [23]	ProGlider: (16/0.02); PathFile: (16/0.02)	20/40	Tempered Steel	90°/5 mm	Synthetic oil (Super Oil; Singer Co. Ltd., Elizabethport, NJ, USA)	NCF FL	ProGlider: 300 rpm; PathFile: 300 rpm	
Gambarini et al., 2015 [24]	K-file (15) in M4 handpiece; PathFile (16/0.02)	10/20	SS	60°/5 mm		TF	K-File: 1250 rpm; PathFile: 100 rpm	
Capar et al., 2015 [25]	PathFile: (16/0.02); G File: (12/0.03); ScoutRaCe: (15/0.02); HyFlex GPF: (15/0.02); ProGlider: (16/0.02)	10/100	SS	90°/3 and 5 mm	Special oil (WD-40 Company, Milton Keynes, UK)	NCF FL	PathFile: 300 rpm; ProGlider: 300 rpm; HyFlex GPF: 300 rpm; G File: 400 rpm; ScoutRaCe: 800 rpm	Air (23 ± 2 °C)
Uslu et al., 2016 [26]	ProGlider (16/0.02); OneG (14/0.03)	20/40	SS	60°/5 mm	Synthetic lubricant (WD Company, Milton Keynes, UK)	NCF FL	ProGlider: 300 rpm; OneG: 400 rpm	
Kwak et al., 2016 [27]	OneG (14/0.03) OneG (14/0.03)—with heat treatment; ProGlider (14/0.03); ProGlider (14/0.03)—with heat treatment	2/10	Tempered Steel	90°/3 mm	Synthetic oil (WD-40; WD-40 Company, San Diego, CA, USA)	TF	OneG: 300 rpm; ProGlider: 300 rpm	
Yılmaz et al., 2017 [28]	ProGlider (16/0.02); OneG (14/0.03); Hyflex EDM (10/0.05)	20/60	Artificial canal (not specified)	Coronal curvature: 60°/5 mm	Synthetic lubricant (WD-40 Company, Milton Keynes, UK)	NCF FL	ProGlider: 300 rpm/200 gcm^−1^ torque; OneG: 300 rpm/1.2 gcm^−1^ torque; Hyflex EDM: 300 rpm/1.8 gcm^−1^ torque	
				Apical curvature: 70°/2 mm				
Topçuoğlu et al., 2018 [29]	R-Pilot (12.5/0.04); WaveOne Gold Glider (15/0.02–0.06)	30/60	SS	45° or 60°/ 5 mm	Oil (WD-40 Company, Milton Keynes, UK)	TF FL	R-Pilot: “RECIPROC” program; WOGG: “WAVEONE GOLD” program	
Serefoglu et al., 2018 [30]	R-Pilot; WaveOne Gold Glider; ProGlider	10/30	SS	90°/3 mm	Synthetic oil (WD-40 Company, Milton Keynes, UK)	NCF FL	R-pilot: “RECIPROC” program/300 rpm; WOGG: “WAVEONE” program/350 rpm; ProGlider: “PROGLIDER” program	
Nishijo et al., 2018 [31]	HyFlex EDM Glide Path File (10/0.05); HyFlex GPF (15/0.02); ScoutRaCe (15/0.02)	20/60	SS 3-pin device	60°/5 mm	Silicone oil	TF	Reciprocating motion (300 rpm); continuous rotation (300 rpm)	
Uslu et al., 2018 [32]	R-Pilot (12.5/0.04); Hyflex EDM (10/0.05); PathFile (19/0.02)	20/60	SS	Coronal curvatue: 60°/5 mm	Synthetic lubricant (WD-40 Company, Milton Keynes, UK)	NCF FL	R-Pilot: “Reciproc ALL” program; HyFlex EDM: 300 rpm/1.8 N·cm torque; PathFile: 300 rpm/3 N·cm torque	
				Apical curvature: 70°/2 mm				
Özyürek et al., 2018 [33]	R-Pilot; WaveOne Gold Glider	20/40	SS	60°/5 mm	Water	TF FL	R-Pilot: “Reciproc ALL” program WOG Glider: “WaveOne ALL” program	Intracanal temperature (35 °C)
Yılmaz et al., 2018 [34]	OneG (14/0.03); ProGlider (16/0.02); HyFlex EDM (10/0.05); R-Pilot (12.5/0.04)	20/80	SS	60°/5 mm	Water	TF FL	OneG: 300 rpm/1.2 N·cm torque; ProGlider: 300 rpm/4 N·cm torque; HyFlex EDM: 300 rpm/1.8 N·cm torque; R Pilot: “Reciproc ALL” program	Body temperature (35 °C)
Keskin et al., 2018 [35]	R-Pilot (12.5/0.04); ProGlider (16/0.02); WaveOne Gold Glider (15/0.02–0.06)	15/45	SS	60°/5 mm	Synthetic oil (WD-40; Milton Keynes, UK)	TF FL	ProGlider: 300 rpm/500 g cm^−1^ torque; R-Pilot: “Reciproc ALL” program; WaveOne Gold Glider: “WaveOne ALL” program	
Topcuoglu et al., 2018 [36]	PathFile (16/0.02); ProGlider (16/0.02–0.08); ScoutRaCe (15/0.02)	30/90	SS	Coronal curvature: 60°/5 mm	Oil (Super-Oil; Singer, Elizabethport, NJ, USA)	NCF FL	ProGlider: 300 rpm/3 N·cm^−1^ torque; PathFile: 300 rpm/3 N·cm^−1^ torque; ScoutRaCe: 800 rpm/1 N·cm^−1^ torque	
				Apical curvature: 70°/2 mm				
Lee et al., 2019 [37]	ProGlider (16/0.02); OneG (14/0.03); EdgeGlidePath (16/progressive taper)	15/45	SS	90°/3 mm		NCF FL	ProGlider: 300 rpm; OneG: 300 rpm; EdgeGlidePath: 300 rpm	
Kırıcı et al., 2019 [38]	ProGlider (16/0.02–0.085); PathGlider (15/0.03); OneG (14/0.03)	20/60	SS	90°/3 mm	Synthetic lubricant (WD-40 Company, Milton Keynes, UK)	TF	ProGlider: 300 rpm/2.5 N·cm; PathGlider: 300 rpm/2.5 N·cm; OneG: 300 rpm/2.5 N·cm	
Kırıcı and Kuştarcı, 2019 [39]	ProGlider (16/0.08); OneG (16/0.06); WaveOne Gold Glider (15/0.08)	15/45	SS	Coronal curve: 60°/5 mm	Water	NCF FL	OneG: 300 rpm/2 N of torque; ProGlider: 300 rpm/2 N of torque; WaveOne Gold Glider: “WaveOne All” program (350 rpm)	
				Apical curve: 70°/2 mm				
Perez-Villalba D et al., 2021 [40]	ProGlider (16/0.02); WaveOne Gold Glider (15/0.02)	25/100	SS	60°/3 mm	3% NaOCl or 3%NaOCl/HEBP 9%	TF	ProGlider: 300 rpm; WaveOne Gold Glider: “WaveOne motion”	Body temperature (37 ± 1 °C)

CFR: cyclic fatigue resistance; TF: time to failure (fracture) in seconds; FL: length of fractured fragment (mm); NCF: number of cycles until failure.

**Table 3:** Significant results from the included studies.

Author, Year	TF(s)		NCF		*p*-Value
Lopes et al., 2012 [21]			PathFile > ScoutRaCe > C-Pilot		<0.05
Sung et al., 2014 [22]			PathFile #1 > PathFile #2 > (PathFile #3, G1) > G2		<0.05
Elnaghy and Elsaka, 2015 [23]			ProGlider > PathFile		<0.001
Gambarini et al., 2015 [24]	K-File connected to M4 handpiece (SybronEndo, Glendora, CA, USA) > PathFile				*p* = 0.033
Capar et al., 2015 [25]			Radius 3 mm	HyFlex GPF > G files > ProGlider > PathFile > ScoutRaCe	From 0.0035 to less than 0.0001
			Radius 5 mm	HyFlex GPF > G files > ProGlider > PathFile > ScoutRaCe	
Uslu et al., 2016 [26]			Pro-Glider ˃ One G		˂0.05
Kwak et al., 2016 [27]	OneGH and ProGliderH > OneG and ProGlider (heat treated > not heat treated); OneG and OneGH > ProGlider and ProGliderH (short pitch > long pitch)				<0.05
Yılmaz et al., 2017 [28]			Double curve	HEDM > ProGlider > OneG	<0.05
			Single curve	HEDM > ProGlider > OneG	
Topçuoğlu et al., 2018 [29]	45° curvature				
	60° curvature	WOGG > R pilot	WOGG > R-Pilot		<0.05
Serefoglu et al., 2018 [30]			WOGG > R-Pilot > ProGlider		<0.05
Nishijo et al., 2018 [31]	Reciprocating motion	HyFlex EDM > GPF > RaCe			<0.05
	Continuous rotation motion	(HyFlex EDM, GPF) > RaCe			
Uslu et al., 2018 [32]			Coronal curvature	R-Pilot > HyFlex EDM > PathFile	<0.05
			Apical curvature	R-Pilot > HyFlex EDM > PathFile	<0.05
Özyürek et al., 2018 [33]	R-Pilot > WOGG				˂0.05
Yılmaz et al., 2018 [34]	R-Pilot > (HyFlex EDM, ProGlider) > OneG				<0.05
Keskin et al., 2018 [35]	(WOGG, R-Pilot) > ProGlider				<0.05
Topcuoglu et al., 2018 [36]			Coronal curve		
			Apical curve	ProGlider > (ScoutRaCe, PathFile)	<0.05
Lee et al., 2019 [37]			EdgeGlidePath > ProGlider > OneG		<0.05
Kırıcı et al., 2019 [38]	ProGlider > (OneG, PathGlider)				<0.001
	OneG > PathGlider				<0.05
Kırıcı and Kuştarcı, 2019 [39]			Coronal curvature	WaveOne Gold Glider > ProGlider > OneG	<0.05
			Apical curvature	WaveOne Gold Glider > ProGlider > OneG	
Perez-Villalba D et al., 2021 [40]					

**Table 4:** Quality assessment.

Author	1	2A	2B	3	4	5	6	7	8	9	10	11	12	13	14	%
Lopes et al., 2012 [21]	Y	Y	Y	Y	Y	N	N/a	N/a	N/a	N/a	Y	Y	N	N	N	64
Sung et al., 2014 [22]	Y	Y	Y	Y	Y	N	N/a	N/a	N/a	N/a	Y	Y	N	N	N	64
Elnaghy and Elsaka, 2015 [23]	Y	Y	Y	Y	Y	N	N/a	N/a	N/a	N/a	Y	Y	N	N	N	64
Gambarini et al., 2015 [24]	Y	Y	Y	Y	Y	N	N/a	N/a	N/a	N/a	Y	Y	N	N	N	64
Capar et al., 2015 [25]	Y	Y	Y	Y	Y	N	N/a	N/a	N/a	N/a	Y	Y	Y	Y	N	82
Uslu et al., 2016 [26]	Y	Y	Y	Y	Y	N	N/a	N/a	N/a	N/a	Y	Y	Y	N	N	73
Kwak et al., 2016 [27]	Y	Y	Y	Y	Y	N	N/a	N/a	N/a	N/a	Y	Y	N	Y	N	73
Yılmaz et al., 2017 [28]	Y	Y	Y	Y	Y	N	N/a	N/a	N/a	N/a	Y	Y	N	N	N	64
Topçuoğlu et al., 2018 [29]	Y	Y	Y	Y	Y	Y	N/a	N/a	N/a	N/a	Y	Y	Y	N	N	82
Serefoglu et al., 2018 [30]	Y	Y	Y	Y	Y	N	N/a	N/a	N/a	N/a	Y	Y	N	N	N	64
Nishijo et al., 2018 [31]	Y	Y	Y	Y	Y	N	N/a	N/a	N/a	N/a	Y	Y	N	Y	N	73
Uslu et al., 2018 [32]	Y	Y	Y	Y	Y	Y	N/a	N/a	N/a	N/a	Y	Y	N	N	N	73
Özyürek et al., 2018 [33]	Y	Y	Y	Y	Y	Y	N/a	N/a	N/a	N/a	Y	Y	Y	N	N	82
Yılmaz et al., 2018 [34]	Y	Y	Y	Y	Y	Y	N/a	N/a	N/a	N/a	Y	Y	Y	N	N	82
Keskin et al., 2018 [35]	Y	Y	Y	Y	Y	N	N/a	N/a	N/a	N/a	Y	Y	N	N	N	64
Topcuoglu et al., 2018 [36]	Y	Y	Y	Y	Y	Y	N/a	N/a	N/a	N/a	Y	Y	Y	N	N	82
Lee et al., 2019 [37]	Y	Y	Y	Y	Y	N	N/a	N/a	N/a	N/a	Y	Y	N	Y	N	73
Kırıcı et al., 2019 [38]	Y	Y	Y	Y	Y	N	N/a	N/a	N/a	N/a	Y	Y	Y	Y	N	82
Kırıcı and Kuştarcı, 2019 [39]	Y	Y	Y	Y	Y	N	N/a	N/a	N/a	N/a	Y	N	N	N	N	55
Perez-Villalba D et al., 2021 [40]	Y	Y	Y	Y	Y	N	N/a	N/a	N/a	N/a	Y	Y	Y	Y	N	82
Mean																72

N: not reported in the article; Y: reported in the article; N/a: does not apply; %: percentage of item compliance per article.

## Data Availability

Additional data can be obtained upon reasonable request from the corresponding author.

## Supplementary Materials

The following supporting information can be downloaded at: https://www.mdpi.com/article/10.3390/ma15196662/s1. Table S1: Search strategy; Table S2: Quantitative results.
