# Supplementary material for: Cyclic Fatigue Resistance of Glide Path Rotary Files: A Systematic Review of in Vitro Studies
Source: Materials (Basel). 2022 Sep 26;15(19):6662. doi: 10.3390/ma15196662 (PMC9571085; doi:10.3390/ma15196662)
Supplement: Supplementary file 1 [file materials-15-06662-s001.zip › Supplementary Table S1.pdf]

Supplementary Table S1. Search strategy

Database	Search strategy	Findings
Medline	#1 All fields (“glide path” OR patency)	46,786
	#2 All fields (“rotary files”)	1480
	#3 All fields (“root canal” OR endod*)	91,598
	#4 All fields (“cyclic fatigue” OR fracture)	344,660
	#1 AND #2 AND #3 AND #4	30
Scopus	#1 TITLE-ABS-KEY (“glide path” OR patency)	1,146
	#2 TITLE-ABS-KEY (“rotary files”)	2,303
	#3 TITLE-ABS-KEY (“root canal” OR endod*)	48,410
	#4 TITLE-ABS-KEY (“cyclic fatigue” OR fracture)	44,948
	#1 AND #2 AND #3 AND #4	12
Embase	#1 (“glide path” OR patency)	64,093
	#2 (“rotary files”)	1,271
	#3 (“root canal” OR endod*)	92,964
	#4 (“cyclic fatigue” OR fracture)	431,516
	#1 AND #2 AND #3 AND #4	24
Web of Science	#1 TS=(“glide path” OR patency)	38,464
	#2 TS=(“rotary files”)	2,328
	#3 TS=(“root canal” OR endod*)	49,240
	#4 TS=(“cyclic fatigue” OR fracture)	623,273
	#1 AND #2 AND #3 AND #4	79
SciELO	#1 (“glide path” OR patency)	30
	#2 (“rotary files”)	60
	#3 (“root canal” OR endod*)	11,113
	#4 (“cyclic fatigue” OR fracture)	1,678
	#1 AND #2 AND #3 AND #4	0
