# Supplementary material for: Cyclic Fatigue Resistance of Glide Path Rotary Files: A Systematic Review of in Vitro Studies
Source: Materials (Basel). 2022 Sep 26;15(19):6662. doi: 10.3390/ma15196662 (PMC9571085; doi:10.3390/ma15196662)
Supplement: Supplementary file 1 [file materials-15-06662-s001.zip › Supplementary Table S2.pdf]

Supplementary Table S2. Quantitative results

Author	Files		TF (s) (Mean ± standard deviation of TF)	NCF	FL (mm)
Lopes et al. ,2012 [21]	Engine-driven C pilot files			237,5 ± 22,8	5,85
	PathFile			645 ± 28,9	6,53
	Scout RaCe			451,5 ± 9,5	7,56
Sung et al ,2014 [22]	G1			314 ± 32	6.6 ± 0.2
	G2			155 ± 13	6,6 ± 0,2
	PathFile #1			386 ± 43	6,4 ± 0,2
	PathFile #2			347 ± 41	6,7 ± 0,2
	PathFile #3			293 ± 31	6,7 ± 0,3
Elnaghy & Elsaka ,2015 [23]	ProGlider			490,35 ± 34,30	6,54 ± 0,21
	PathFIle			360,45 ± 29,04	6,62 ± 0,27
Gambarini et al. ,2015 [24]	K-File		1049 ± 24,8		
	PathFile		464 ± 40,4		
Capar et al. ,2015 [25]	PathFile	Radius 3 mm		512 ± 82	4,03 ± 0,16
		Radius 5 mm		733 ± 104	4,16 ± 0,13
	G-File	Radius 3 mm		873 ± 147	4,09 ± 0,26
		Radius 5 mm		1803 ± 417	4,07 ± 0,18
	Scout Race	Radius 3 mm		372 ± 80	4,14 ± 0,23
		Radius 5 mm		588 ± 88	4,24 ± 0,32
	HyFlex GPF	Radius 3 mm		2059 ± 602	4,23 ± 0,22
		Radius 5 mm		12887 ± 1851	4,15 ± 0,12
	ProGlider	Radius 3 mm		656 ± 92	4,20 ± 0,29
		Radius 5 mm		1056 ± 114	4,14 ± 0,16

Uslu et al. ,2016 [26]	Pro-Glider				25,082 ± 6009,37	6,2 ± 0,2
	One G				21,316 ± 4169,90	5,7 ± 0,2
Kwak et al. ,2016 [27]	OneG				71,4 ± 9,0	
	OneGH				194,7 ± 24,4	
	pG				46,2 ± 8,7	
	pGH				170,3 ± 24,4	
Yılmaz et al. ,2017 [28]	Pro Glider	Double curve	Coronal curve		1.020,28 ± 375,34	6,03 ± 1,05
			Apical curve		961,87 ± 293,45	2,11 ± 0,56
		Single curve			1.614,65 ± 601,42	4,93 ± 1,02
	One G	Double curve	Coronal curve		470,21 ± 152,7	6,72 ± 1,67
			Apical curve		395,63 ± 139,05	2,08 ± 0,34
		Single curve			846,94 ± 274,96	5,43 ± 1,39
	Hyflex EDM	Double curve	Coronal curve		2.458,32 ± 538,65	6,68 ± 1,39
			Apical curve		1.966,46 ± 412,43	2,24 ± 0,64
		Single curve			3.441,24 ± 961,84	5,25 ± 1,22
Topçuoğlu et al. ,2018 [29]	R-PILOT	45° Curve		394,5 ± 45,3	2185,45 ± 255,16	5,01 ± 1,08
		60° Curve		247,2 ± 36,2	1355,58 ± 328,41	5,32 ± 1,21
	WaveOne Gold Glider	45° Curve		412,4 ± 55,2	2304,36 ± 346,21	5,12 ± 0,98
		60° Curve		368,3 ± 44,1	2116,37 ± 424,64	5,23 ± 1,36
Serefoglu et al. ,2018 [30]	R-Pilot				1038 ±177	6,8 ± 0,34
	WaveOne Gold Glider				1294 ± 123	7,1 ± 0,21
	ProGlider				266 ± 72	6,9 ± 0,28
Nishijo et al. ,2018 [31]	HyFlex EDM	Reciprocating motion		**1650		
		Continuous rotation		**700		

	Glide Path File				
	HyFlex GPF	Reciprocating motion	**1300		
		Continuous rotation	**500		
	Scout RaCe	Reciprocating motion	**250		
		Continuous rotation	**100		
Uslu et al. ,2018 [32]	R-Pilot	Coronal curve		4894,82 ± 743,11	7,65 ± 1,44
		Apical curve		3607,57 ± 519,06	2,11 ± 0,29
	HyFlex EDM	Coronal curve		2262,43 ± 271,44	7,76 ± 1,34
		Apical curve		1706,72 ±209,72	2,19 ± 0,44
	PathFile	Coronal curve		1675,41 ± 201,55	7,84 ± 1,45
		Apical curve		1062,03 ± 127,44	2,12 ± 0,35
Özyürek et al. ,2018 [33]	R-Pilot		1073.55 ± 139.49		5,42 ± 0,41
	WaveOne Gold Glider		846.73 ±126.91		5,38 ± 0,36
Yılmaz *et al. ,2018* [34]	One G		193,68 ± 26,42		5,23 ± 0,31
	ProGlider		329,41 ± 48,49		5,33 ± 0,34
	HyFlex EDM		388,21 ± 46,62		5,01 ± 0,32
	R-Pilot		915,71 ± 173,96		5,09 ± 0,29
Keskin et al. ,2018 [35]	R-Pilot		3562,46 ± 963,55		4.45 ± 0.46
	ProGlider		1254,20 ± 356,08		4.63 ± 0.27
	WaveOne Gold Glider		3465,26 ± 468,54		4.67 ± 0.43
Topcuoglu et al. ,2018 [36]*	PathFile	Coronal curve		537,3 (297,6–912,3)	5,27 ± 1,63
		Apical curve		326,4 (211,8–532,1)	2,21 ± 0,58

	ProGlider	Coronal curve		604,5 (389,4–1024,5)	5,68 ± 1,27
		Apical curve		517,3 (347,3–924,5)	2,37 ± 0,49
	ScoutRaCe	Coronal curve		565,2 (303,5–964,6)	5,02 ± 1,46
		Apical curve		349,6 (237,4–659,3)	2,63 ± 0,73
Lee et al. ,2019 [37]	ProGlider			3568 ± 282	2,82 ± 0,17
	One G			1529 ± 192	2,21 ± 0,42
	EdgeGlidePath			5079 ± 714	2,33 ± 0,99
Kırıcı et al. ,2019 [38]	ProGlider		101		
	PathGlider		63		
	One G		73		
Kırıcı & Kuştarcı ,2019 [39]	ProGlider	Coronal curve		1.331,75 ± 65,41	6,98 ± 0,13
		Apical curve		666,25 ± 34.97	2,14 ± 0,1
	One G	Coronal curve		778,3 ± 124.62	6,99 ± 0,19
		Apical curve		487,92 ± 76.11	2,16 ± 0,7
	WaveOne Gold Glider	Coronal curve		1.868,08 ± 55,16	7,02 ± 0,09
		Apical curve		1.482 ± 57,54	2,15 ± 0,11
Perez-Villalba D et al. ,2021 [40]	ProGliderG & NaOCl				
	ProGlider & NaOCl/HEBP				
	WOGG & NaOCl/HEBP				
	WOGG & NaOCl				

*: Data provided as median (Min-Max); **: Only provides approximate data through a graph; TF (s): Time to fracture (in seconds); NCF: Number of cycles to fracture; FL (mm): Length of fractured fragment
